# Differentiation of *Rhodnius neglectus* and *Rhodnius prolixus* (Hemiptera: Reduviidae: Triatominae) by multiple parameters

**DOI:** 10.1590/0037-8682-0503-2019

**Published:** 2020-04-03

**Authors:** Rossana Falcone, Aline Rimoldi Ribeiro, Jader de Oliveira, Vagner José Mendonça, Márcia Graminha, João Aristeu da Rosa

**Affiliations:** 1 Departamento de Ciências Biológicas, Universidade Estadual Paulista, Araraquara, SP, Brasil.; 2 Departamento de Biologia Animal, Universidade Estadual de Campinas, Campinas, SP, Brasil.; 3 Departamento de Parasitologia e Microbiologia, Universidade Federal do Piauí, Teresina, PI, Brasil.; 4 Departamento de Análises Clínicas, Universidade Estadual Paulista, Araraquara, SP, Brasil.

**Keywords:** Taxonomy, Phylogeny, Cytochrome b, Morphology, Geometric morphometrics

## Abstract

**Introduction:**

The genus *Rhodnius* in the subfamily Triatominae comprises 20 species, which can transmit *Trypanosoma cruzi* and *Trypanosoma rangeli*. Due to the development of molecular techniques, Triatominae species can now be characterized by mitochondrial and nuclear markers, making it possible to verify and/or correct the existing data on these species. The results achieved in this study provide a more detailed and accurate differentiation of the *Rhodnius* species, helping the establishment of a more appropriate classification.

**Methods:**

Data collection was performed by DNA analysis, morphological and morphometric studies to distinguish four populations of *R. neglectus* and four of *R. prolixus*. Phylogenetic data were compared to morphological and morphometric data.

**Results:**

The analysis of Cytb fragments suggests that the four colonies designated to *Rhodnius neglectus* as well as those of *R. prolixus* were correctly identified.

**Conclusions:**

The morphological characters observed in the specimens of the colonies originally identified as *R. prolixus* and *R. neglectus*, such as the presence or absence of collar in the eggs, the patterns of the median process of the pygophore, and anterolateral angle, are consistent with the species. Geometric morphometrics also show an intraspecific variability in *R. prolixus*.

## INTRODUCTION

Chagas disease is caused by *Trypanosoma cruzi* and is estimated to infect 6 to 7 million people worldwide, mostly in Latin America. The transmission occurs mainly by contact with feces of Triatominae species^1^. 

Currently, there are 154 described species of triatomines distributed in 19 genera and grouped into five tribes^2^ ^,^ ^3^ ^,^ ^4^ ^,^ ^5^ ^,^ ^6^. The species are described and classified based on traditional morphology-based taxonomy that recently evolved to incorporate new types of data, such as molecular details^7^ ^,^ ^8^ ^,^ ^9^, which has been very useful in supporting phylogenetic relationships and assisting with taxonomic issues^10^. 

The genus *Rhodnius* comprises 20 species of potential *Trypanosoma cruzi* and *T. rangeli* vectors and is considered one of the taxonomically complex genera in Triatominae^11^ ^,^ ^12^ ^,^ ^13^. The morphological similarities and geographical distribution (which may overlap in some *Rhodnius* species) make it difficult to study such species^11^ ^,^ ^12^ ^,^ ^13^. Various studies have adopted approaches to achieve a more accurate description and classification of different *Rhodnius* species and their interrelations, such as eggs, male and female genitalia, and ribosomal DNA^14^ ^,^ ^15^ ^,^ ^16^.

Triatomines of the genus *Rhodnius* are among the most difficult to identify, especially species of the complex *R. prolixus* (*R. prolixus*, *R. robustus*, *R. neglectus* and *R. nasutus*), due to morphological similarity among them. Incorrect identification results in data loss, hindering the orientation of anti-vector actions^12^.

*Rhodnius prolixus* Stal, 1859 is the main vector of the Chagas disease in some areas of Northern South America, being found in sixteen Latin American countries^17^. 

In countries like Venezuela and Colombia, *R. prolixus* is considered sylvatic, mainly inhabiting the crowns of multiple species of palm trees, but in some areas it can be found already well established in human dwellings^18^ ^,^ ^19^ ^,^ ^20^. In 2011, all the previously endemic countries of Central America had been formally certified as free of their main domestic vector, *R. prolixus* ^21^.

*Rhodnius neglectus* Lent, 1954 is considered a wild species and is largely distributed in Brazil (in Federal District and other 13 states: Acre, Bahia, Goiás, Maranhão, Mato Grosso, Mato Grosso do Sul, Minas Gerais, Paraíba, Pernambuco, Paraná, Piauí, São Paulo, Tocantins), being characteristic of the Cerrado biome. There are no occurrence records of *R. neglectus* outside the Brazilian territory where it has often invaded human habitations, with a secondary role in the transmission of Chagas disease^10^ ^,^ ^22^.

In the São Paulo state territory, during the period between 1990 and 1999, 3,149 specimens of *R. neglectus* were collected, of which 2,542 were from inside dwellings and 607 in the peridomicile. Specimens of *R. neglectus* were also found colonizing palm trees and invading buildings in the Araçatuba and Monte Alto areas of São Paulo state^23^ ^,^ ^24^.

In this work, morphological, morphometric and molecular tools were used to provide a more detailed differentiation of *R. neglectus* and *R. prolixus*, which could contribute to taxonomic, phylogenetic, and ecological studies.

## METHODS

### Objects of study

The *Rhodnius* colonies used in this study are maintained at the Triatomine Insectarium of the São Paulo State University (UNESP), School of Pharmaceutical Sciences, Araraquara.

Four populations of *R. neglectus* and four of *R. prolixus* were studied (Table 1):

**TABLE 1:** *Rhodnius* colonies used: nomenclature, point of collection and starting date.

CODE	Colony of Triatominae of Araraquara	Species	Origin	Starting date
A	CTA 61	*R. neglectus*	Frutal/SP/Brazil - Sylvatic (coconut palms)	05/1983
B	CTA 62	*R. neglectus*	Frutal/SP/ Brazil - Peridomestic (chicken coop)	05/1983
C	CTA 65	*R. neglectus*	Frutal/SP/Brazil - Domestic	08/1983
D	CTA 229	*R. neglectus*	Formoso/GO/Brazil - *	06/2011
E	CTA 73	*R. prolixus*	Panama, El Salvador and Costa Rica - *	05/1983
F	CTA 74	*R. prolixus*	Venezuela - Sylvatic	05/1983
G	CTA 78	*R. prolixus*	Colombia - *	06/1982
H	CTA 81	*R. prolixus*	Colombia - *	03/1982

*no further information.

### Phylogenetic analysis

Genomic DNA was extracted from the modified second triatomine protocol by Bargues and Mas-Coma^25^. The tissue of choice was the leg muscles, which guarantee the absence of contaminating microorganisms.

The primer used in the amplification of the cytochrome b gene (Cytb) has been described by Monteiro et al*.* ^26^ *.* (Forward: 5’ GGACG(AT)GG(AT)ATTTATTATGGATC 3’ and Reverse: 5’ GC(AT)CCAATTCA(AG)GTTA(AG)TAA 3’), with annealing temperature set at 45ºC. The PCR (Polymerase Chain Reaction) was performed in a BIO-RAD T100 thermal cycler. PCR protocol was performed following the Thermo Scientific for the High Fidelity PCR Enzyme Mix, including temperatures and concentrations. 

The PCR product was purified using the kit “NucleoSpin® Gel and PCR Clean-Up” (Macherey-Nagel) according to the manufacturer’s instructions for subsequent sequencing. The sequencing of the PCR product was processed by the Technology Department of Faculty of Agrarian and Veterinary Sciences UNESP, Jaboticabal, SP, Brazil.

Phylogenetic analysis was performed using the Mega 6.0 program with the Neighbor-joining statistical method, which generates dendrograms based on the genetic distance of the organisms, the length of the branches representing the proportion of divergent nucleotides. The Bootstrap phylogenetic test was performed with 1000 replications, considering values above 75% as well supported^13^.

### Morphological analysis

***Eggs:*** The macroscopic observations in this study comprised the presence or absence of a close binding to the operculum of the eggs called the “collar”, described by Forattini & Barata^27^ and considered a character that differentiates *R. neglectus* from *R. prolixus*. To observe this characteristic, 30 eggs were used, and the images captured with a stereoscopic microscope Leica M205C by 1x Leica Planapo 10450028 lens through the LAS 4.12.0 software.

### Median process of the pygophore (male genitalia)

A protocol approached by Eunice Galatti in 2015^28^ was adapted to mount the anatomic sample (pygophore). First, one male was taken from each colony and their genitalia were removed, then, placed in a porcelain crucible in the presence of potassium hydroxide 10%. After 24 hours, the parts were separated into parameres, phallus, and median process of the pygophore then returned to the container for an additional 12 hours for clarification. Alcohol dehydration process was carried out by placing the samples in 70%, 90%, 95%, and absolute alcohol for 10 minutes then drying at room temperature. The dried pieces were immersed in Eugenol for 3 hours and then mounted between a slide and cover slip in the presence of Canada balsam. The drying time of the slides at room temperature was approximately five days.

### Anterolateral angle

For the analysis of the anterolateral angles, images of structures located on the pronotum of 10 specimens of each colony were collected using a stereo microscope Leica M205C by 1x Leica Planapo 10450028 lens through the LAS 4.12.0 software, then the shape of the anterolateral angle was observed. The structure is prominent in *R. neglectus*, whereas it has a rounded shape and comparatively not as prominent in *R. prolixus* ^7^ ^,^ ^10^. 

### Geometric morphometrics analysis

To analyze the geometric morphometrics, images of the head of 15 specimens were captured by means of a stereo microscope Leica MZ APO with an increase of 20x through the New Capture 2.0 program. Using the CLIC 98 program, eight anatomical landmarks selected from Gurgel-Gonçalves et al*.* ^12^ were applied to each image. The analysis of the collected coordinates was also made using the CLIC program 98 through an orthogonal method of projections called Procrustes to adjust the figures to common points, i.e., translation to a common centroid, scaling to the same centroid size, and rotation to minimize summed squared distances between the corresponding landmarks**.**

**FIGURE 1:**
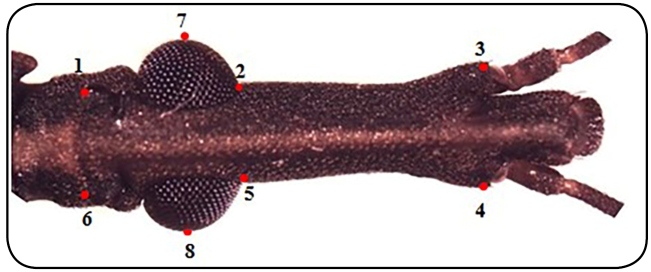
*Rhodnius neglectus* head showing the eight landmarks selected for geometric morphometrics. Points 1, 2, 5 and 6 are Type I (tissue juxtaposition) and points 3, 4, 7 and 8 are Type II (maximum curvature)^11^.

The discriminant analysis was performed using the major components of Procrustes. A factorial map was built with the first two principal components (“eigenvalues” CP1 and CP2) to observe the existence of differences between *R. neglectus* and *R. prolixus* (Figure 1).

## RESULTS

### Phylogenetic analysis

The Cytb gene sequences were manually set then the junction was performed with all sequences, including those selected and collected from GenBank®, and finally aligned.

**FIGURE 2:**
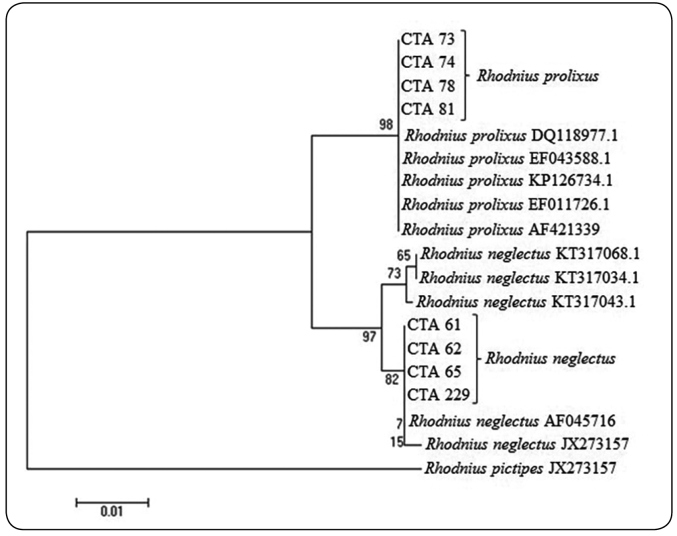
Phylogenetic dendrogram from colonies of *R. neglectus* and *R. prolixus* for gene fragment Cytb based on Neighbor-joining analysis. Sequences related to CTAs studied with addition of sequences available at GenBank.

The dendrogram was constructed using the Neighbor-joining statistical method along with Bootstrap and rooted with an outgroup of *R. pictipes*. The results generated from the analysis of the Cytb gene were inserted in the clade samples corresponding to colonies 73, 74, 78 and 81 along with the sequences of *R. prolixus* collected at GenBank® (Table 2). The samples related to colonies 61, 62, 65, and 229, corresponding to *R. neglectus*, remained grouped with the sequences of *R. neglectus* obtained from GenBank®. This clade is further from the other in evolutionary terms (Figure 2).

**TABLE 2:** Access codes of sequences from the mitochondrial gene Cytb collected at GenBank®.

Species	Access code	Species	Access code
*Rhodnius neglectus*	AF045716	*Rhodnius prolixus*	EFO11726.1
*Rhodnius neglectus*	JX273156.1	*Rhodnius prolixus*	KP126734.1
*Rhodnius neglectus*	KT317043.1	*Rhodnius prolixus*	EFO43588.1
*Rhodnius neglectus*	KT317034.1	*Rhodnius prolixus*	DQ118977.1
*Rhodnius neglectus*	KT317068.1	*Rhodnius prolixus*	AF421339
*Rhodnius pictipes*	JX273157		

### Morphological analysis

**FIGURE 3:**
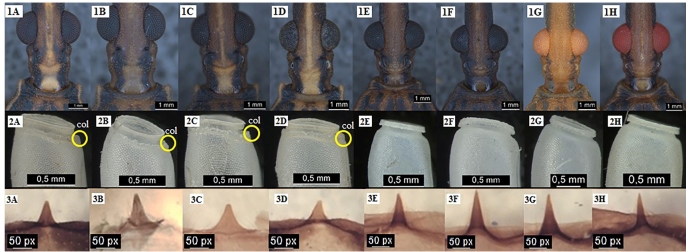
Anterolateral angles of *R.* neglectus: 1A-CTA 61, 1B-CTA 62, 1C-CTA 65 and 1D-CTA 229. *R. prolixus*: 1E-CTA 73, 1F-CTA 74, 1G-CTA 78 and 1H-CTA 81. Eggs of *R. neglectus* from colonies 2A-CTA 61, 2B-CTA 62, 2C-CTA 65 and 2D-CTA 229; eggs of *R. prolixus* from colonies 2E-CTA 73, 2F-CTA 74, 2G-CTA 78 and 2H-CTA 81 (col. = collar). Median process of the pygophore in *R. neglectus*: 3A-CTA 61, 3B-CTA 62, 3C-CTA 65, 3D-CTA 229 and *R. prolixus*: 3E-CTA 73, 3F-CTA 74, 3G-CTA 78 and 3H-CTA 81.

***Presence or absence of collar:*** According to Forattini & Barata^27^, a macroscopic means for differentiating R*. prolixus* from *R. neglectus* is the presence or lack of an agglutination next to the lid, called *collar*. The collar was noticed in all *Rhodnius prolixus* colonies, which followed the pattern described for the species showing no existence of the collar (Figure 3).

### Median process the pygophore (male genitalia)

Comparing the male genitalia images presented by Jurberg^29^ with the images from the colonies studied in this work, it was observed that colonies 73, 74, 78, and 81 have a similar pattern to the median process of the pygophore described for *R. prolixus* in the chapter mentioned above by Jurberg^29^. Colonies 61, 62, 65, and 229 show a pattern that resembles the one described as belonging to *R. neglectus* species^26^(Figure 3).

### Anterolateral angle

The pattern of anterolateral angles in colonies *of R. neglectus* and *R. prolixus* was observed (Figure 3). While in *R. neglectus* such angles are prominent, in *R. prolixus,* the structure has a more rounded shape and is not so prominent^10^ ^,^ ^30^. Based on the patterns found by the authors, colonies 61, 62, 65, and 229 correspond to the *R. neglectus* pattern, and colonies 73, 74, 78 and 81 match *R. prolixus* pattern.

### Geometric morphometrics

**FIGURE 4:**
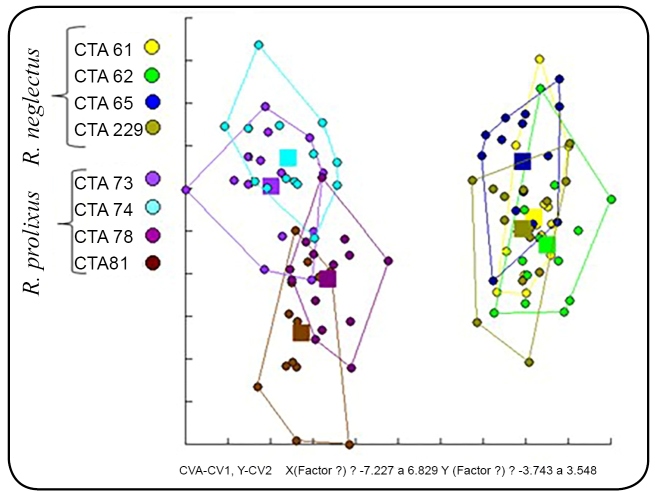
Factorial map of discriminant analysis of the head of four colonies of *R. neglectus* and four of *R. prolixus*.

The factorial map in Figure 4, generated by discriminant analysis, reveals the formation of two distinct groups of colonies, the one on the right consisting of colonies 61, 62, 65 and 229, and the one on the left gathering colonies 73, 74, 78 and 81. Intraspecific variability can be noticed in both species. Factor 1 was responsible for a variation of 10.14%, with a greater influence on the construction of the factorial map, whereas factor 2 range was 6.88%. 

## DISCUSSION

Nowadays, the study of triatomines is important because, despite all control measures by public health surveillance, vector transmission still represents approximately 80% of Chagas disease cases^31^. The misidentification of *Rhodnius neglectus* and *R. prolixus* also generates a lot of controversies on the dispersion of the species in Brazil and raises questions about its occurrence in the country. Dias et al.^19^ consider that *R. prolixus* occurs in the states of Amazonas and Tocantins, whereas Galvão^6^ and Jurberg et al.^14^contend that the species is not present in Brazil.

This study has made a multiparametric analysis to assess the effectiveness of a taxonomic differentiation using only one parameter or more especially since it is difficult to find parameters that can precisely differentiate the species of the complex *R. prolixus*. Barrett^32^, for example, described the genus *Rhodnius* as consisting of species without a clear interspecific limit and with many morphological similarities among themselves, especially the complex *R. prolixus*. Evaluation of the results in this study shows that molecular and geomorphometric parameters were particularly effective in distinguishing *R. neglectus* from *R. prolixus*.

Abad-Franch et al.^33^ showed four species having genetic distance between *R. prolixus* and *R. neglectus* in phylogenetic analysis using the Cytb gene,*,* which is in agreement with the results obtained in this study. Studying many species, Lyman et al.^34^ pointed out the possibility to separate *R. neglectus* and *R. prolixus* using Cytb, also demonstrating the existence of genetic distance between them, a result that is in accordance with the ones achieved in this study.

Monteiro et al.^35^ also drew attention to the need to search for useful characters for identification of the complex *R. prolixus*. In view of this need, morphological parameters described in the literature are discussed, verifying their effectiveness on the studied species. Forattini & Barata^27^ suggested the differentiation between *R. neglectus* and *R. prolixus* by features found in their eggs, one of the main parameters being the presence or absence of collar, which is observed in this work for separation of the colonies. The morphological difference pointed out by Lent and Wygodzinsky^7^ was also valid in this study.

Jurberg et al.^36^ considered the male genitalia as an important factor in the differentiation among species of the genus *Rhodnius*. In this study, the median process of the pygophore, which is part of the male genitalia, was used to distinguish four colonies of *R. neglectus* and five of *R. prolixus*. It was noted that there are two patterns among the colonies studied consistent with *R. neglectus* and *R. prolixus*. Soares et al.^13^ have drawn attention to the need of using other parameters along with the male genitalia in cases where differentiation among the species *R. prolixus* is complex, but in this study this technique proved effective. 

Gurgel-Gonçalves et al*.* ^12^ discuss the importance that geometric morphometrics has acquired in recent years to solve taxonomic problems, which led to the decision to include this promising tool in this work. The outcome of this study reinforces the value of this parameter in the differentiation of *R. neglectus* from *R. prolixus*. The results contradict Dujardin et al*.* ^37^, who point out that geometric morphometrics head would be minor in comparison with the wing as a general measure of morphological differentiation. However, the outcomes were in accordance with Gurgel-Gonçalves et al*.* ^12^, who regard geometric morphometrics, including the head parameter, as a useful method for separating similar *Rhodnius* species.

This study generated a lot of molecular data, of which geomorphometric and morphological data of specimens of *R. neglectus* and *R. prolixus* maintained in the laboratory can be used as a basis for comparison with field colonies or other colonies maintained in the laboratory. In the development of this work, it was observed that all the four populations of*R. neglectus* and the four of*R. prolixus*have intraspecific morphological and morphometric variability (Figure 3 and Figure 4), despite being held in the laboratory for a long time. 

The results discussed here indicate that the identification of colonies is more consistent and reliable when different parameters are used together.
